# From Doubt to Diagnosis: Canadian Patient Perspectives on a Limb‐Girdle Muscular Dystrophy Diagnosis

**DOI:** 10.1111/hex.70271

**Published:** 2025-05-05

**Authors:** Homira Osman, Zainab Adamji, Gerald Pfeffer, Jodi Warman‐Chardon, Pryamvada Varma, Jenna Keindel, Stacey Lintern

**Affiliations:** ^1^ Muscular Dystrophy Canada Toronto Canada; ^2^ Neuromuscular Disease Network of Canada Ottawa Canada; ^3^ Hotchkiss Brain Institute, Department of Clinical Neurosciences, Cumming School of Medicine University of Calgary Calgary Canada; ^4^ Alberta Child Health Research Institute, Department of Medical Genetics, Cumming School of Medicine University of Calgary Calgary Canada; ^5^ Department of Medicine (Neurology) The Ottawa Hospital/The University of Ottawa Ottawa Canada; ^6^ Faculty of Medicine/Eric Poulin Centre for Neuromuscular Disease University of Ottawa Ottawa Canada; ^7^ Ottawa Hospital Research Institute Ottawa Canada

**Keywords:** diagnostic odyssey, limb‐girdle muscular dystrophy, neuromuscular disease, patient journey, patient perspective, rare disease

## Abstract

**Introduction:**

Limb‐girdle muscular dystrophies (LGMDs) encompass a rare and genetically diverse set of disorders, posing challenges in diagnosis due to the absence of distinct pathological features, leading to frequent misdiagnoses and inadequate symptom management. Yet, there is a scarcity of published data on how patients perceive the diagnostic journey of LGMD. Our aim was to unveil the firsthand experiences of individuals with LGMD to gain insight into their perspective on the diagnosis process. This study comprehensively captures the LGMD patient and caregiver experiences—from symptom onset through diagnosis to current disorder management.

**Methods:**

Insights into the lived experience of LGMD were consolidated from semi‐structured interviews and a cross‐sectional mixed‐methods survey of quantitative and qualitative questions. Quantitative data were analysed using descriptive statistics and frequencies, while inductive content analysis was applied to qualitative responses. During the validation phase, patient authors validated and prioritised the insights and overarching themes.

**Results:**

From 108 participants (104 people with LGMD and 4 parent caregivers), five overarching themes were identified. These themes include (1) difficulty with diagnostic process, with 8 years noted as time from the symptom onset until they obtain the definitive diagnosis; (2) difficulty obtaining genetic testing and specialist care; (3) sense of disconnect with healthcare professionals, often resulting from lack of knowledge and awareness of the condition; (4) a state of emotional distress, feelings of hopelessness, depression, fear and anxiety with the diagnosis process; and (5) impact on mobility and ambulation.

**Conclusion:**

The LGMD diagnosis journey is marked by barriers and misdiagnoses, leading to considerable diagnostic delays. Overcoming these challenges requires increased awareness among healthcare professionals and improved patient access to genetic testing.

**Patient or Public Contribution:**

Patients with LGMD were involved as research partners in all phases of this study, including identifying the research question and the need for an assessment of the diagnosis journey for LGMD in Canada. The patients also worked with the authors to interpret and validate the data collected and contributed to the preparation of the manuscript by participating in the review and editing process.

## 1 Introduction

Limb‐girdle muscular dystrophies (LGMDs) are a diverse group of rare genetically inherited neuromuscular diseases encompassing several subtypes [1]. Clinically, LGMD manifests as progressive weakness in the hip and shoulder girdle muscles [2, 3, 4]. LGMDs as a group have an estimated prevalence of 1–6 per 100,000 people [1, 5], with more than 30 associated genes [6, 7]. Each of the genes associated with LGMD encodes specific proteins residing within the muscle cell and is responsible for muscle function, repair and regulation [4, 8]. Severity, rate of progression, age of symptom onset and systemic organ involvement can vary and contribute to heterogeneous experiences. Yet, LGMD patients frequently encounter distinct challenges with exercise, irregular walking (gait) patterns (i.e., tip‐toe walking and walking on the balls of feet), and elevated serum creatine kinase (CK) levels [6, 9]. A diagnosis of LGMD is typically made by a neurologist using genetic testing with support from genetics staff (e.g., medical geneticists and genetic counsellors). In Canada, while genetic testing largely resides in its hospitals, testing for LGMD is commissioned to out‐of‐country laboratories and may not be covered by provincial and territorial health systems.

Other investigations, such as muscle biopsy, serum CK levels, neurophysiology, muscle ultrasound and magnetic resonance imaging, can be useful as supportive evidence for the diagnosis but are often non‐specific. There are currently no cures for LGMD; therefore, the treatments available are generally geared towards the management of symptoms and prevention of complications [10].

The clinical and histological features of LGMD often overlap with other neuromuscular conditions, including inflammatory muscle disease like necrotising myopathies [11, 12, 13, 14]. The classic pathological features for LGMD may also be absent in some patients, due to patchy muscle involvement, causing sampling error on muscle biopsy as well as preferentially affecting certain muscles [15], or may be no longer visible in end‐stage muscle tissue [16]. This can make differentiating LGMD from other myopathies (e.g., metabolic or myofibrillar myopathies) much more challenging. The progressive nature of the disease and presentation of non‐specific symptoms often make patient diagnosis challenging [1]. Furthermore, despite the availability of genetic testing, some patients live for years without receiving a confirmed diagnosis [1]. Receiving a definite genetic diagnosis is an important part of the diagnostic journey as it provides patients and their caregivers with information on prognosis, access to appropriate supportive treatment plans to manage symptoms and disease progression and access to clinical trials.

There are few qualitative studies describing the impacts of LGMD on patients and specifically reporting the diagnostic journey in patients with LGMD. In one study, common themes regarding the impact on patients with an LGMD diagnosis included mobility limitations, inability to participate in daily and social activities, weakness and negative perceptions around body image [6]. Concerns around disease progression and future health, a lack of awareness of LGMD amongst healthcare professionals (HCPs) and feelings of emotional distress were reported in a study, which emphasised the importance of holistic person‐centred care in patients diagnosed with LGMD [3]. This demonstrates that the impacts transcend the physical symptoms experienced by patients with LGMD.

There have been no studies in Canada that describe the diagnostic journey experienced by patients living with LGMD grounded in the patient voice. This study provides both quantitative and qualitative evidence about the LGMD patient experience from symptom onset to receiving a diagnosis and current management of the disease. Exploring the patient‐reported impacts of receiving an LGMD diagnosis is important, as each experience is unique and can contribute towards building awareness and supporting advocacy efforts towards accessing timely diagnoses, particularly through genetic testing.

## 2 Methods

Twelve individuals with a confirmed LGMD diagnosis who had completed ‘imPORTND Patient‐Oriented Research Training’ were invited to participate in 1‐h‐long semi‐structured interviews conducted virtually. The interviews were conducted using a pre‐defined framework designed to capture three main lines of enquiry: (1) the patient's journey: experiences from symptom onset to diagnosis; (2) interactions with healthcare professionals: positive and challenging aspects; and (3) impact on life: effects on daily activities, relationships and emotional well‐being.

This framework helped organise responses, while flexibility in the semi‐structured format allowed the inclusion of additional insights beyond these categories. The analysis of the interviews focused on identifying participant experiences (e.g., delays in diagnosis and emotional challenges), unique insights that reflected variations in individual journeys and recurring barriers or enablers in healthcare interactions.

A mixed‐methods survey was designed based on the three categories outlined in the framework. It comprised 17 quantitative questions and 21 qualitative questions with free‐text response options. The survey was co‐developed in collaboration with three patient partners (Canadian adults with confirmed LGMD diagnoses). The self‐report survey addressed the following areas.

### 2.1 The Patient's Journey

- Disease‐related information: Initial symptoms, top bothersome symptoms, progression of disease, perceived impact of symptoms and diagnosis, family history of LGMD and specific type of LGMD (if known).
- Temporal information: Time to medical care from initial symptoms, time to diagnosis from initial medical care and time to management.
- Patients' perceptions of information on LGMD (i.e., the individual's feeling of their own level of knowledge of the disease and resources and support available).
- Patients' main source of disease information: Participants were asked to identify their key sources for disease information at the time of symptom onset, diagnosis and at present.

### 2.2 Interactions With Healthcare Professionals

- Diagnosis‐related information: Number of physicians visited before the patient received a diagnosis, incorrect initial diagnoses and reasons for any delayed contact with specialist care from initial symptoms.
- Patients' perceptions of HCPs and the clinical care team's knowledge of LGMD and interactions with HCPs in diagnosis and care.
- Patients' perceptions on the diagnostic process: barriers to genetic testing, initial feelings and questions on genetic test results at the time of diagnosis, understanding of genetic diagnosis, satisfaction and level of stress with the overall diagnostic process.

### 2.3 Impact on Life

Patients' perceptions on everyday life and reflections on improving diagnosis and quality of life with LGMD.

### 2.4 Participants

Adult participants 18 years of age and older were invited to participate in the survey through email and social media communications through a national patient advocacy organisation, Muscular Dystrophy Canada (MDC), from July to September 2023. Purposive sampling of individuals with LGMD registered with MDC was used as a recruitment strategy to identify participants to complete the online survey. Inclusion criteria included membership in MDC and a clinical diagnosis of LGMD or caregivers of children with a confirmed diagnosis of LGMD living in Canada. Consent was obtained from participants before interview and survey completion. Every participant (100%) was confirmed to have a clinical diagnosis of LGMD according to their MDC registration form, which was approved by a neurologist or neuromuscular specialist.

### 2.5 Survey Analysis

Twenty survey responses were excluded from analysis for reasons including (1) a clinical diagnosis of LGMD or membership with MDC was not confirmed and (2) survey responses included limited information such as name, age and province. Quantitative responses were summarised using descriptive statistics and reported as means and ranges where applicable. Free‐text responses were analysed using conventional content analysis [17] with an interpretivist approach by two authors independently identifying recurring concepts within the data, organised under the initial three categories. Sub‐concepts were then derived from manifest content, which refers to the visible and obvious components. The focus was on extracting and reporting the descriptive level of content rather than delving into deeper interpretations or underlying meanings.

During the validation phase, the patient authors evaluated the sub‐concepts related to the patient's journey, interactions with healthcare professionals and impact on life. The team members engaging in direct interactions with study participants considered how their professional backgrounds and association with a patient advocacy organisation could have affected data interpretation, or the participants' willingness to candidly discuss their experiences, and the content of their disclosures.

## 3 Results

### 3.1 Participant Demographics

A total of 128 participants attempted the survey, including the 12 individuals who had previously participated in the semi‐structured interviews. Twenty survey responses were excluded from analysis for reasons including (1) a clinical diagnosis of LGMD or membership with MDC was not confirmed and (2) survey responses included limited information such as name, age and province. Therefore, this study had a final sample size of 108, of which 104 were people with a confirmed diagnosis of LGMD and 4 were parents of children and adolescents with a LGMD diagnosis. There were 61 female and 43 male participants. The average age of participants with LGMD was 34.96 years (2–85 years), and the average age of caregivers was 44.71 years (34–57 years).

### 3.2 Patient Journey

#### 3.2.1 Symptom Onset

Participants reported that their initial symptoms were often generalised: muscle weakness, fatigue, weight loss, discomfort and pain, and a decrease in strength and function. The most frequently reported initial symptom was noticeable difficulty with stairs/getting up off the ground, followed by muscle pain or weakness, lack of stamina and increased fatigue, frequent falls and resultant fear of falling, decreased ability to participate in physical activities and occasional ‘tip‐toe’ walking. Participants sought medical attention 4 years following their first bothersome symptom (Table 1), primarily because of three factors: physical symptoms became bothersome, a positive family history and recommendations from third parties such as family members, rheumatologists, chiropractors or physiotherapists. The majority experienced a sudden onset of symptoms, with only 17 out of 96 participants noting a gradual progression of their symptoms over time. Due to insufficient public awareness or information about neuromuscular diseases, participants struggled to comprehend the reasons behind their initial symptoms and indicated that LGMD rarely surfaced as a conceivable diagnosis in their considerations.

I had to get back to school after long summer vacation (which I spent at home mostly), and I quickly recognized something was wrong with me—I could no longer walk for a long distance confidently, use stairs and run easily like I did before.

Person with LGMD

**Table 1:** Participants' demographics within the symptom onset phase.

Symptom onset	
Mean age at symptom onset (years)	21 (2–62)
Length of time from bothersome symptom onset and initial HCP visit (years)	4 (0.03–25)
Touchpoints consulted before first healthcare visit	
Parents	43 (30%)
Family members	22 (15%)
Partner	26 (18%)
Friends	20 (14%)
Teacher/Academic professional	3 (2%)
Nurse/School nurse	1 (< 1%)
Nobody	23 (16%)
Other	5 (4%)
Type of HCP visited at symptom onset	
Family doctor/general practitioner	68 (70%)
Neurologist	20 (21%)
Other (specialist doctors/ER)	8 (8%)
No one	1 (1%)

Fifteen out of 96 participants mentioned that recognising a genetic predisposition to LGMD motivated them to seek a neurologist referral. Additionally, 19 out of 96 participants were directed to specialists for diagnostic evaluation based on observations of weakness by family members, with majority referred to neurologist based on elevated CK levels.

My brother came home from college and noticed I was starting to walk funny. A teacher also asked my parents if I was hurt because I looked like I was struggling to run in gym.

Person with LGMD

### 3.3 Patient Interactions With Healthcare Professionals

#### 3.3.1 Information Seeking

During the initial symptom onset, participants reported seeking information from sources including the internet, scientific journals, textbooks, organisations like Muscular Dystrophy Canada, their HCPs and family members with a confirmed diagnosis.

When asked about the participants' knowledge of LGMD, where 0 meant not knowledgeable and 100 meant very knowledgeable, participants rated their knowledge of LGMD as 12 out of 100. During the diagnosis process, 44 out of 89 participants stated that they were given information about LGMD when they received a definitive diagnosis. Participants utilised comparable resources for information at the time of diagnosis as they did during the onset of symptoms.

#### 3.3.2 Diagnostic Odyssey

In this study, the average wait to get a confirmed diagnosis was 8 years from the onset of initial symptoms (Table 2). During this period of awaiting an accurate diagnosis, 28 out of 96 participants received at least one incorrect diagnosis, and 36 out of 96 participants received two or more inaccurate diagnoses. Thirteen out of 96 participants reported that their doctors told them that their symptoms were psychological/behavioural (i.e., laziness, poor health habits/sedentary and ‘it's all in your head’) in nature. Eleven out of 96 participants were told they had a muscle/inflammatory myopathy. Participants also received misdiagnoses such as polymyositis, chronic weakness/fatigue syndrome, Duchenne/Becker muscular dystrophies, facioscapulohumeral muscular dystrophy (FSHD), rheumatoid arthritis and amyotrophic lateral sclerosis.

**Table 2:** Participants' demographics with diagnosis and everyday life with LGMD.

Diagnosis	
Family history of LGMD	
Yes	15 (16%)
No	71 (74%)
I don't know	10 (10%)
Age at diagnosis (years)	28 (1–65)
Length of time from bothersome symptom and confirmed diagnosis (years)	8 (0–34)
Confirmed genetic testing	
Yes	65 (73%)
No	15 (17%)
Don't know	9 (10%)
LGMD subtype	
Type 1A (myofibrillar myopathy)	3 (3%)
Type 1B (Emery–Dreifuss)	1 (< 1%)
Type 1C (Rippling muscle disease)	2 (2%)
Type 2A	19 (21%)
Type 2B	13 (15%)
Type 2C	2 (2%)
Type 2D	4 (6%)
Type 2H	2 (2%)
Type 2I	11 (12%)
Type 2L	3 (3%)
Type 2T	1 (< 1%)
Unsure/Don't know	22 (25%)
Other	6 (7%)
Everyday Life with LGMD	
Current supportive treatments (physical and occupational therapy, rehabilitation, assistive devices, orthoses and supplements)	
Yes	22 (25%)
No	65 (75%)

In certain cases, participants noted a cascading effect in the diagnosis process, leading to an extended diagnostic journey. For instance, some received a misdiagnosis of Becker muscular dystrophy simply because family members had previously received the same misdiagnosis. Only after additional testing did they confirm their actual diagnosis as LGMD.

I was told, “Your creatine kinase (CK) level is high. Since your brother has Becker muscular dystrophy, you must have it too.” I lived with this misdiagnosis for 17 years, and discovering it was incorrect was truly devastating.

For numerous years, I navigated through uncertainty and confusion. There was no familial history for muscular dystrophy or any neuromuscular disorder. Initially, they suspected polymyositis. Subsequent test results were inconclusive, they described me as being “in the grey zone.” By age 24, the diagnosis shifted to FSHD, and only after further testing was it rectified to LGMD.

#### 3.3.3 Genetic Testing

The majority (85%) of the participants in this study reported that their families did not have a history of LGMD. While all had a confirmed diagnosis of LGMD, 73% obtained a confirmed diagnosis through genetic testing (Table 2). During the diagnostic process, participants outlined a series of tests that typically began with a blood test to validate elevated CK levels, proceeded to a muscle biopsy and generally concluded with a genetic test. Regarding the accessibility of getting this series of tests, 62% encountered some degree of difficulty, and 65% characterised the process as stressful.

Participants were asked to rate various aspects of their testing experience and their satisfaction with the diagnosis process (Table 3).

**Table 3:** Genetic testing and diagnosis experiences.

Testing experience		
	(*N*) %	
	Poor/satisfactory	Good/excellent
Access to testing (proximity to testing facility and availability of appointments)	33 (37)	41 (46)
Mode of testing	27 (30)	43 (48)
Access to timely diagnosis results	31 (34)	37 (41)
Presence of any testing side effects	19 (21)	38 (42)
Cost of testing	13 (15)	56 (63)

Satisfaction with the diagnosis experience			
	**(*N*) %**		
	**Dissatisfied**	**Satisfied**	**Neither satisfied/dissatisfied**
Overall diagnostic process	18 (16)	37 (42)	36 (40)
Information given at diagnosis	22 (19)	38 (43)	29 (35)
Manner of the professional disclosing the diagnosis	16 (18)	51 (58)	28 (24)
Support offered post‐diagnosis	30 (34)	35 (39)	22 (26)

When asked about the barriers experienced during the diagnosis process, participants reported that their HCPs were unwilling to help with obtaining a diagnosis, as they often brushed off symptoms and that they lacked sufficient knowledge about LGMD. Participants also reported that the diagnosis required multiple invasive testing procedures, which only exacerbated their negative experiences, as it also increased the length of time it took to receive a confirmed result. Additionally, some participants had to travel long distances to access their specialists.

My primary care physician got in the way of me getting the right diagnosis. Upon informing him of my diagnosis, he leaned back in his chair and admitted, “I don't have much knowledge about muscular dystrophy.”

Person with LGMD

My biopsies came back unspecified. So, I did not have a confirmed diagnosis for several years. It was through genetic testing conducted outside of Canada that I paid for out of pocket by the way, that I was finally given a confirmed LGMD diagnosis.

Person with LGMD

Some participants received their diagnoses at a young age when information about LGMD was scarce and reported that they were not old enough to fully understand their diagnosis. For other participants, their diagnosis result came as a shock and elicited an overwhelmingly negative response. Throughout the diagnostic process, participants felt dissatisfied with the lack of support and attention that they received from their HCPs. Participants expressed that they perceived their HCPs as dismissive of their symptoms, not taking their experiences seriously. They felt a lack of interest from HCPs in recommending additional testing for a definitive diagnosis. Some participants reported that HCPs conveyed the belief that, without available cures or treatments, pursuing a diagnosis was deemed unnecessary.

For years, I felt dismissed by doctors, often told it's in my head or that my condition was way less severe than I was presenting it to be. It was only when I finally received a genetic diagnosis that I felt they gave me the attention I deserved but you know what, I still struggle with [my neurologist] listening to me ask about research or drugs I read about.

Person with LGMD

The LGMD diagnostic journey can evoke a range of emotions, constituting a challenging period for many. After testing, 17 out of 78 participants reported distressing feelings of hopelessness, depression, fear and anxiety. Conversely, 16 out of 78 participants expressed a sense of relief. Despite the challenges of the diagnosis, confirmation served as an informational tool, empowering them to manage their symptoms, seek clinical trials and review emerging research.

LGMD was the presumptive diagnosis for 20 years. I was very relieved to have genetic confirmation.

Person with LGMD

Following the diagnosis, 92% of individuals expressed acceptance of the diagnosis. Fifty‐one out of 87 participants noted that they would have wanted to receive an earlier diagnosis. Their primary inquiries to neuromuscular specialists, ranked in order of most frequently reported, included questions about prognosis, life expectancy, the inheritance pattern in LGMD, potential impact on other family members, symptom management, progression rate, and available clinical trial or research opportunities—essentially, the pressing question of ‘how close are we to a cure for LGMD?’ Of note, 66 out of 87 participants were not receiving any treatments for LGMD. There were 12 out of 87 participants having physiotherapy and 10 out of 87 of the participants take supplements such as creatine and vitamins.

### 3.4 Impact on Life

#### 3.4.1 Perceptions of LGMD on Everyday Life

The symptoms of LGMD had a wide‐reaching impact on various aspects of participants' lives, affecting them physically, emotionally and socially. Participants observed that the physical limitations imposed by LGMD significantly impacted their mobility and daily activities. Moreover, the diagnosis of LGMD led to unfavourable vocational and psycho‐social consequences, including scepticism and disbelief from other members of society when they disclosed their symptoms.

I was employed as a repair technician with a telephone company. Initially, I managed as best as I could, but the challenges became progressively more difficult. Eventually, I had to inform my employer, which was challenging because, to outward appearances, it seemed like there was “nothing wrong with me,” and I sensed that my fellow employees held that same view—it was like my LGMD was seemingly invisible to them.

Person with LGMD

Participants reported the most significant impact of LGMD in everyday life is on their relationships and personal development. Subsequently, 11 out of 96 participants indicated that they had to forgo social activities, with 24 out of 96 participants having to discontinue sporting activities due to their inability to match the pace and performance of their peers. Participants stressed that their reduced involvement in social activities had adverse effects on their mental well‐being, leaving them with feelings of isolation.

Participants also reported that limitations in mobility accompanied by fatigue resulted in a lack of independence, inaccessible environments limited participants' ability to navigate outdoor spaces, and a lack of support from the government and HCPs led to financial insecurity.

Participants reflected on their life with LGMD and identified five aspects they wished they had to enhance their health experience, listed in order of frequency: (1) informed and knowledgeable healthcare professionals, (2) access to trained specialists including neurologists, geneticists, respirologists and physiatrists as well as wrap‐around support services such as adjustment counselling and peer support groups and services like those offered by organisations like MDC (e.g., assistive devices, financial aid and finding accessible housing), (3) an accurate diagnosis at the onset of symptoms and earlier in the diagnostic process, (4) more information about LGMD at the time of diagnosis and (5) information on current treatments and participation in clinical trials.

I had to make a career change 3 times; knowing more [about LGMD] would have helped me find a job I can do a little longer in my years. I am 52 on disability support struggling to survive because the cost versus the amount you receive is a joke.

Person with LGMD

Lastly, when participants were asked to provide newly diagnosed Canadians with any advice, four key messages emerged. The first was to ‘be positive’, one participant noted:

Over time you will figure out the best way to achieve a task. Everything just takes time and you will learn every day how to deal with it. Have faith in yourself, you're stronger than you think you are.

Person with LGMD

Participants emphasised the need to educate others about LGMD and advocate, which was illustrated by one participant who reported:

Life can still be good. People may judge you. They may ask you if you “injured your back in a car accident” because of the way you move. Instead of wanting to run away and cry, educate them. Education and awareness are how change can happen to make life even better.

Person with LGMD

The importance of maintaining physical health, particularly through diet and some form of activity, was highlighted by participants.

Take good care of your body, this is the body you have been given and this is what has happened with our genetics. When you live a cleaner life, it really makes a difference. It's worthwhile taking care of yourself and diet. Really take care of your body—my genetics are a loaded gun but my daily life is the trigger so don't pull the trigger. Push through the tough times.

Person with LGMD

The fourth key message revolved around asking for support, while participants noted that supports overall were lacking throughout their diagnosis process, they recognised the importance of asking for social supports when it was needed to alleviate what was perceived to be a significant psychologically distressing journey.

My advice would be to ask a social worker or doctor what you can access that would make your life easier. For example, income supports, therapy, etc. It's hard to transition from the unknown to the known mentally, as well as having to adjust your life to what you now know you have. Having support during that is essential.

Person with LGMD

## 4 Discussion

This study aimed to show the diagnostic journey of individuals with LGMD in Canada through a holistic patient‐centred perspective. The findings depict the prolonged diagnostic odyssey and underscore the difficulties inherent in accessing specialist care, testing and obtaining a definitive LGMD diagnosis and navigating life with this rare neuromuscular condition that impacts mobility and psychosocial well‐being.

The 8‐year delay in obtaining a diagnosis can be attributed to the non‐specific symptoms associated with LGMD, as well as the lack of knowledge about the disease among HCPs. This is consistent with the existing literature, as one study investigating the diagnostic delay in patients diagnosed with LGMD type 2I reported that the median diagnostic delay in their cohort was 6.5 years [18]. The primary cause of the delay in the previous study was that patients and their family members did not recognise the significance of their symptoms, and since the symptoms presented varied, it was difficult to attribute them to a specific disease. Misdiagnosis played a significant role in the diagnostic delay observed in this study, with participants reporting alternative diagnoses such as polymyositis and Duchenne/Becker muscular dystrophy. This occurred due to the overlapping clinical presentations. A growing body of evidence indicates that inflammatory myopathies frequently resemble LGMD, emphasising the risks associated with misdiagnosis [11, 12, 19, 20]. For example, one study presented a case where a patient was misdiagnosed with an inflammatory myopathy and offered immunosuppressive therapy; however, the diagnosis was confirmed to be LGMD almost 10 years later [12]. Another report showed how genetic disease may be considered only late in the diagnostic process, if the symptoms are first suspected to be due to a pre‐existing health condition [21].

The current study complements work that demonstrates the heterogeneous nature of the disease in terms of symptom onset, severity and disease progression, and the challenges associated with accessing and interpreting genetic testing contribute to the diagnostic odyssey for LGMD [19, 22]. The most common misdiagnosis for patients in our study was psychological/behavioural factors in 13% of the participants. This emphasises a need for further awareness and physician education. Currently, when patients present with symptoms that do not have an apparent organic cause, the preferred diagnosis is functional neurologic disorder (FND) rather than ‘psychological/behavioural’. The considerations of FND are helpful for the diagnostic process, because it should give physicians pause to determine whether they have truly ruled out an organic cause for the symptoms, or whether further testing or specialist referral should be considered [23].

Factors related to social determinants of health, including income, social status, education level, literacy, gender and race/ethnicity, can exacerbate and influence healthcare accessibility and the availability of testing. Additionally, these factors may introduce provider biases into the consideration of differential diagnoses [24, 25]. These emphasise the importance of a thorough multi‐disciplinary and clinical assessment of symptoms during the diagnostic process and to provide care that is socially and culturally responsive.

A major barrier in obtaining an accurate LGMD diagnosis for the majority (62%) of participants was the difficulty in accessing genetic testing or accurate interpretation of genetic testing results. There is a wide range of differential diagnoses of LGMD that require a DNA analysis to provide an accurate molecular diagnosis, as other investigations do not offer the same specificity [22, 26, 27]. However, accessing molecular testing is complex and expensive and limited by specific provincial reimbursement for genetic testing [28]. This study emphasised the importance of receiving a genetic diagnosis as it allows people with LGMD to participate in clinical trials for therapeutic treatments designed for specific genetic variants, such as in the case of Duchenne muscular dystrophy [29]. In the current study, participants expressed feeling frustrated and apathetic because even in the absence of treatment, they were not being afforded the right to receive accurate knowledge of their diagnosis. A precise genetic diagnosis is key in guiding genetic counselling, disease management, accessing clinical trials and monitoring for disease complications [30].

Given the challenges of genetic testing interpretation, patients with autoimmune muscle diseases can be misdiagnosed with LGMD and miss a crucial period of treatment to limit irreversible weakness [11, 12]. In this study, 14% of participants reported receiving a diagnosis based on their physical symptoms and elevated CK levels, but received an inconclusive genetic test result. This is consistent with the literature in that even with DNA sequencing, genetic variants in patients presenting LGMD phenotypes are only revealed in 20%–45% of the cases [31, 32]. Most patients with slow progressive muscle weakness are typically presumed to have a hereditary disease like LGMD, which was the case in this study. However, there is a growing body of evidence to suggest that autoimmune myopathies such as anti‐HMGCR myopathy may mimic the symptoms of LGMD, which was also reported by some participants in the current study [32, 33]. In one study, 6 out of the 11 participants were presumed to have LGMD based on elevated CK and onset of muscle weakness for almost 10 years, as their genetic tests were unrevealing, and after antibody testing, they were revealed to have anti‐HMGCR myopathies [32]. The authors highlighted the importance of follow‐up testing in patients who are suspected of a LGMD diagnosis but receive inconclusive genetic test results.

The findings of this study underscore the limited knowledge of LGMD throughout the health journey and the imperative for increased awareness and education regarding LGMD. Participants noted that the new nomenclature associated with subtypes of LGMD caused confusion. In fact, one study describing the initiative to change the established terminology for LGMDs addressed this challenge, stating that patient organisations felt that if the terminology were to change, it should be followed up with appropriate education [34]. Patient advocates indicated that these changes could lead to misunderstandings amongst patient communities and non‐experts [34]. Additionally, participants in the present study felt that even at the time of their diagnoses, their HCPs were not well equipped with the knowledge about LGMD to support them. In a study describing the perceptions of young adults receiving an LGMD diagnosis, participants noted that their diagnosis was difficult to comprehend and that they were not offered sufficient information to cope with the disease [35]. The participants in Aho et al.'s [35] study emphasised the need for resources on mental health supports from their doctors as well as information about the disease. Similarly, participants from the current study reported a lack of access to support groups, awareness about LGMD and resources on LGMD. Another study reported that patients living with LGMD felt that there was a lack of knowledge about their disease amongst general practitioners, which resulted in them having to constantly advocate for the support they needed [36]. The patients in that cohort experienced difficulties in contacting HCPs and described feelings of being neglected and not being seen or understood within the healthcare system [36]. These findings highlight the significant diagnostic challenges for patients with rare NMDs [6, 18, 37, 38].

To ensure individuals with LGMD receive necessary support from healthcare providers, there is a heightened necessity to actively listen to patients and incorporate patient partners in studies. A comprehensive understanding of the patient journey relies on the involvement of patient partners, shaping both the study design and the interpretation of results. A recent review highlights the importance of having patient partners in research as their contributions enhance research relevance and design [39]. Patient partners are pivotal in developing research studies that have profound impacts on the community as they provide lived experience input that can help translate findings into practice [40]. Patient involvement additionally facilitates collaboration and offers improved health outcomes for the community at large [39].

This study is the first to portray the diagnostic journey of individuals with LGMD in Canada, actively engaging patient research partners with lived expertise in both the study design and analysis. This approach guarantees an accurate depiction of the diagnostic challenges associated with LGMDs and ensures the prominence of the patient perspective. Knowledge of patient experiences navigating the diagnostic odyssey for LGMD is valuable as it highlights the importance of accurate diagnosis and well‐informed healthcare providers, as well as barriers that must be addressed, such as access to genetic testing. The unique insights gained from this study necessitate further exploration through additional person‐centred research. Notably, a considerable portion of the survey participants in this study were adults with LGMD. A limitation of this study lies in the absence of representation for the experiences of children and caregivers of children. There is a critical need to investigate the diagnostic journey for children and adolescents. Furthermore, the criteria for participation in this study included having a membership with MDC, which may have limited the sample of people with LGMD who were eligible to participate, as well as those who were in the process of receiving a LGMD diagnosis. Future studies will require patient‐centred research to help identify specific processes that would improve the patient experience.

## Author Contributions

**Homira Osman:** conceptualisation, investigation, funding acquisition, writing – original draft, methodology, validation, visualisation, writing – review and editing, formal analysis, project administration, data curation, supervision, resources. **Zainab Adamji:** writing – original draft, validation, writing – review and editing, formal analysis, investigation. **Gerald Pfeffer:** writing – review and editing, formal analysis. **Jodi Warman‐Chardon:** writing – review and editing, formal analysis. **Pryamvada Varma:** writing – review and editing, validation, visualisation. **Jenna Keindel:** validation, visualisation, writing – review and editing. **Stacey Lintern:** validation, visualisation, writing – review and editing, funding acquisition, conceptualisation, supervision, resources.

## Consent

Informed consent was obtained from all individual participants included in this study.

## Conflicts of Interest

The authors declare no conflicts of interest.

## Data Availability

The data that support the findings of this study are available on request from the corresponding author. The data are not publicly available due to privacy or ethical restrictions, as this is a rare neurological disease in Canada.
